# Supplementary material for: Health and social care workers experiences of coping while working in the frontline during the COVID-19 pandemic: One year on
Source: PLoS One. 2023 Apr 12;18(4):e0284306. doi: 10.1371/journal.pone.0284306 (PMC10096225; doi:10.1371/journal.pone.0284306)
Supplement: Attachment — Submitted filename: Response to Reviewers.docx [file pone.0284306.s002.docx]

Dr Jo Billings

UCL Division of Psychiatry

6^th^ Floor, Maple House

149 Tottenham Court Road

London W1T 7NF

Muhammad Arsyad Subu
Academic Editor
PLOS ONE

3^rd^ March, 2023

Dear Dr Subu

**LONDON’S GLOBAL UNIVERSITY**

Thank you to you and your reviewer for your feedback on our manuscript **Health and social care workers’ experiences of coping while working in the frontline during the COVID-19 pandemic: one year on (PONE-D-22-34685).**

We have provided a point-by-point response to yours and the reviewer’s comments in the table below.

| Comments from Academic Editor | | Page |
| --- | --- | --- |
| 1. Please ensure that your manuscript meets PLOS ONE's style requirements, including those for file naming. | We have carefully checked PLOS ONE’s style requirements and amended heading font sizes etc to make sure that the manuscript meets these requirements throughout.  We have renamed the Supplementary File to meet these requirements | -  Supplementary materials |
| 2. In your Methods section, please include additional information about your dataset and ensure that you have included a statement specifying whether the collection and analysis method complied with the terms and conditions for the source of the data. | Additional information about the dataset is provided in the results section, such as the participant sociodemographic information represented in Table 1.  We have added a statement in the method section about the collection and analysis method compliance after we described how this was done. | Results, p10  Method, p7 |
| 3. We note that you have indicated that data from this study are available upon request. PLOS only allows data to be available upon request if there are legal or ethical restrictions on sharing data publicly. For more information on unacceptable data access restrictions, please see <http://journals.plos.org/plosone/s/data-availability#loc-unacceptable-data-access-restrictions>.  In your revised cover letter, please address the following prompts:  a) If there are ethical or legal restrictions on sharing a de-identified data set, please explain them in detail (e.g., data contain potentially sensitive information, data are owned by a third-party organization, etc.) and who has imposed them (e.g., an ethics committee). Please also provide contact information for a data access committee, ethics committee, or other institutional body to which data requests may be sent.  b) If there are no restrictions, please upload the minimal anonymized data set necessary to replicate your study findings as either Supporting Information files or to a stable, public repository and provide us with the relevant URLs, DOIs, or accession numbers. For a list of acceptable repositories, please see <http://journals.plos.org/plosone/s/data-availability#loc-recommended-repositories>.  We will update your Data Availability statement on your behalf to reflect the information you provide. | We have clarified this in the Data Availability section by removing the sentence “Data can be made available upon reasonable request to the corresponding author”.  a) The original data from this study cannot be made available upon request because of the personal and sensitive nature of the health and social care workers accounts. Audio recordings of the interviews were destroyed once they were transcribed, which is in line with ethical considerations/approval from the both the UCL and Royal Holloway Research Ethics Committees.  To enquire about data requests, contact the UCL Research Ethics Committee on [ethics@ucl.ac.uk](mailto:ethics@ucl.ac.uk) | Declarations, p48 |
| 4. Please review your reference list to ensure that it is complete and correct. If you have cited papers that have been retracted, please include the rationale for doing so in the manuscript text, or remove these references and replace them with relevant current references. Any changes to the reference list should be mentioned in the rebuttal letter that accompanies your revised manuscript. If you need to cite a retracted article, indicate the article’s retracted status in the References list and also include a citation and full reference for the retraction notice. | We have reviewed the references and noticed there was an error in the order of the papers. This has now been corrected and reference numbers that were changed have been highlighted.  We have not included any cited papers that have been retracted, all have been mentioned in the paper. | - |
| Comments from Reviewer | | |
| Abstract Comment: Please follow the guidelines in writing your abstract. | We have reviewed the abstract and edited it according to the guidelines outlined on the PLOS ONE website- https://journals.plos.org/ plosone/s/submission-guidelines.  We did this by reducing the word count from 301 to 298 and by removing 2 abbreviations. | Abstract, p2 |
| Interviewer/facilitator Comment: Which author/s conducted the data collection? What experience or training did the researcher have? | We have now stated which authors took part in data collection and detailed their training and experience. | Method- Study design, Participants and Procedure, p8 |
| Study design Comment: • Research Ethics Committee (Ref. 18341/001) in page 6 should be put in ethical considerations section (page 7). • Grounded theory as the methodological orientation must be stated in this study design section. Please describe this design (Charmaz constructivist GT). I suggest authors to provide a brief explanation of research paradigm or worldview of this adopted GT. | The information on the Research Ethics Committees has now been moved to the ethical considerations section.  We have added a paragraph at the beginning of the study design, participants and procedure subsection to state that grounded theory was the methodological orientation and a brief explanation of why this was adopted. | Method- Ethical Considerations, p8  Method- Study design, Participants and Procedure, p6 |
| Data collection Comments: • For interview guide, was it pilot tested or not? • The duration of the interviews should be put in this section. Please remove the duration of interviews in results section. • In what interview was data saturation achieved? • Were transcripts returned to participants for comment and/or correction? | • The interview schedule was not pilot tested as this would not be considered usual practice in qualitative interviews, especially when using grounded theory. In grounded theory, questions will evolve with every new participant and whenever there is new insight after an interview. It is expected that changes be made to the interview schedule to cater for new lines of enquiry that might come up after an interview.  • Thank you for the suggestion to move the duration of the interviews from the results to the methods section. However, we would prefer to keep this information in the results section as is usual practice when reporting on qualitative research interviews achieved.  • We did not use the data saturation approach to determine sample size. While we appreciate that data saturation is a method that has been used in grounded theory, we preferred to use the ‘information power’ approach as data saturation has been criticised (Braun and Clarke, 2021; Malterud et al., 2016). We have now added a paragraph outlining our choice in approach and explaining the decision to use the ‘information power’ approach instead of data saturation. Apologies for not including this in the first manuscript.  • We did not send the transcripts to participants routinely. We sent one transcribed interview to a participant to invite further feedback, but they did not provide further feedback.  All interviews were audio recorded. They were then transcribed verbatim by the researcher conducting the interviews and checked against the audio recording for accuracy by other members of the research team.  We endeavoured to improve the validity of our analyses by sharing our findings with individuals with lived experience (a healthcare worker and a social care worker) and researchers/experts in the field. | -  -  Method- Study design, Participants and Procedure, p8  - |
| Data analysis Comments: Clear (Charmaz’ GT -- constructive data analysis).  Study rigor: It is important that study rigor needs to be explained after data analysis (P9) just before your reflexivity. | We have added a paragraph on study rigor at the end of the Analysis section, before the Reflexivity section. In this paragraph we explained how we adhered to the Standards of Reporting Qualitative Research (SRQR) outlined by O’Brien and colleagues (O’Brien et al., 2014). | Method- Analysis, p10 |
| Data and ﬁndings and reporting Comments: I suggest that authors indicate each category separately (category 1, category 2, etc.) | Thank you for the suggestion, which was discussed with the research team.  After due consideration, the team felt that the current subheadings were more appropriate and most accessible. | - |
| I suggest authors to adopt Consolidated criteria for reporting qualitative research (COREQ): Tong A, Sainsbury P, Craig J. Consolidated criteria for reporting qualitative research (COREQ): a 32-item checklist for interviews and focus groups. International Journal for Quality in Health Care. 2007. Volume 19, Number 6: pp. 349 – 357. Available at: <https://academic.oup.com/intqhc/article/19/6/349/1791966> | Thank you for your suggestion. While preparing the report we adhered to the Standards of Reporting Qualitative Research (SRQR) outlined by O’Brien and colleagues (O’Brien et al., 2014). The SRQR is a list of 21 items that are considered essential for complete and transparent reporting of qualitative research. Apologies for not mentioning this in the first manuscript. | Method- Analysis, p10 |

Thank you again for considering our paper. We hope these amendments address the points raised and very much look forward to hearing further from you.

With kind regards

**Dr Jo Billings**

**Consultant Clinical Psychologist**

**Associate Clinical Professor, Division of Psychiatry, UCL.**


**References**

Braun & Clarke (2021) To saturate or not to saturate? Questioning data saturation as a useful concept for thematic analysis and sample-size rationales. Qualitative Research in Sport, Exercise and Health, 13, 2, 201-216. doi:10.1080/2159676X.2019.1704846

Malterud, Siersma & Guassora (2016). Sample Size in Qualitative Interview Studies: Guided by Information Power. Qualitative Health Research. 26, 13, 1753-1760. doi:10.1177/1049732315617444

O'Brien, B. C., Harris, I. B., Beckman, T. J., Reed, D. A., & Cook, D. A. (2014). Standards for reporting qualitative research: a synthesis of recommendations. Academic medicine : journal of the Association of American Medical Colleges, 89(9), 1245–1251. https://doi.org/10.1097/ACM.0000000000000388
